# Feasibility of accelerated partial breast irradiation with volumetric-modulated arc therapy in elderly and frail patients

**DOI:** 10.1186/s13014-015-0516-3

**Published:** 2015-10-14

**Authors:** Olivier Riou, Pascal Fenoglietto, Céline Bourgier, Olivier Lauche, Fatiha Boulbair, Marie Charissoux, Angélique Ducteil, Norbert Aillères, Claire Lemanski, David Azria

**Affiliations:** Radiation Oncology Department, Institut régional du Cancer de Montpellier (ICM), Val d’Aurelle, 208 avenue des Apothicaires, 34298, Montpellier, cedex 5 France; Radiotherapy Department, Mulhouse Hospital, Mulhouse, France

**Keywords:** Intensity-modulated radiotherapy, Arc therapy, Breast cancer, Accelerated partial breast irradiation, Organs at risk

## Abstract

**Background:**

Accelerated partial breast irradiation (APBI) is an option for adjuvant radiotherapy according to ASTRO and ESTRO recommendations. Among the available techniques, volumetric-modulated arc therapy (VMAT) is attractive but has not been extensively studied for APBI. This study assessed its feasibility, tolerance and early oncological outcomes.

**Methods:**

We analysed the data of nine patients (median age 74 years) with ten lesions (one bilateral cancer) treated from May 2011 to July 2012 with APBI using VMAT. The radiation oncologist delineated the surgical tumour bed, and added an 18 mm isotropic margin to obtain the planning target volume (PTV). The dose was 40 Gy prescribed in 4 Gy fractions given twice a day over five days. Patients were regularly followed for toxicities and oncological outcomes.

**Results:**

Mean PTV was 100.0 cm^3^ and 95 % of the PTV received a mean dose of 99.7 % of the prescribed dose. Hot spots represented 0.3 % of the PTV. 6.2 %, 1.6 % and 0.3 % of the ipsilateral lung volume received 5 Gy (V_5Gy_), 10 Gy (V_10Gy_) and 20 Gy (V_20Gy_), respectively. Regarding the contralateral lung, V_5Gy_ was 0.3 %, and V_10Gy_ and V_20Gy_ were nil. V_5Gy_ accounted for 3.1 % of the heart. An average 580 monitor units were delivered. No acute or late grade ≥ 2 toxicities were observed. With a median follow-up of 26 months, no relapses occurred.

**Conclusion:**

In our study, VMAT allowed optimal dosimetry with consequential high therapeutic ratio in elderly and frail patients.

## Background

Breast-conserving therapy followed by whole breast irradiation (WBI) equals radical surgery in terms of overall survival with limited long-term toxicity [1–3]. Even though the role of radiotherapy is well established, its use is sometimes challenged owing to accessibility, equipment and cost issues. Hypofractionated radiotherapy, such as accelerated partial breast irradiation (APBI), could be a response to certain WBI drawbacks. APBI allows to shorten treatment time and to limit the exposure of organs at risk (OAR) with a putative equivalent efficacy compared to standard fractionated WBI in patients with early breast cancer [4–12]. APBI is of special interest in elderly or frail patients who barely tolerate standard-course radiotherapy. A wide variety of APBI techniques are used such as brachytherapy (interstitial needles or balloon-based), intraoperative or external beam radiotherapy, especially 3-dimensional conformal radiotherapy (3D-CRT) [13]. The latter is mainly performed thanks to its availability, but the optimal delivery technique remains to be determined [6]. Recent studies have raised concerns regarding possible higher toxicity rates after APBI. In particular, after balloon catheter brachytherapy, a significant higher risk of rib fracture, breast pain and fat necrosis have been reported [14]. After external beam radiotherapy (intensity-modulated radiation therapy [IMRT] and/or 3D-CRT), a fair aesthetic outcome has also been noted, in specific clinical, technical and dose conditions [15, 16]. On the contrary, another group has published improved toxicity results when using IMRT APBI as compared to WBI plus tumor bed boost [17, 18]. These results call for new clinical studies on other delivery techniques of APBI, which might entail different types and rates of toxicity.

Volumetric-modulated arc therapy (VMAT) is an attractive IMRT technique that enables a fast delivery and an improved efficiency. It has been evaluated in a broad spectrum of tumours but has not been extensively studied for APBI [19–21]. Qiu et al. reported dosimetry feasibility of the VMAT approach compared to 3D-CRT treatment planning in 8 patients with breast cancer and showed that VMAT was more efficient, with equivalent or improved dose conformity and lower doses to OAR [22]. Essers et al. compared VMAT and 3D-CRT in a larger series of 37 patients [23] No clinical data has been yet reported with VMAT APBI. Here, we present the feasibility and early clinical results of BC patients treated with VMAT APBI.

## Methods

### Patient selection

Our study design was validated by our institutional ethical board (Comité d'éthique) and informed consent was obtained from all patients before treatment.

Nine patients with early breast cancer (ten lesions: 5 right-sided, 3 left-sided and 1 bilateral breast cancer) were prospectively recruited from May 2011 to July 2012. Patients’ inclusion criteria were based on the guidelines of both work-task forces ASTRO and GEC/ESTRO for APBI indications [24, 25]. All patients were older than 50 years and had undergone breast-conserving surgery with free margins followed by APBI using VMAT. They all had pT1N0 invasive ductal carcinoma, grade 1–2 SBR score, positive hormonal receptor, and negative HER2 expression. The patients were excluded in case of metastatic disease. Patients’ characteristics are listed in Table 1. All patients were elderly (above 70) or presented with serious comorbidities including cardiovascular disease, chronic respiratory insufficiency or major scoliosis.

**Table 1:** Patients characteristics

Patient no.	Age (years)	T stage	N Stage	Tumour size (mm)	HR	HER2	SBR	Treatment side	Tumour location	Sequential adjuvant chemotherapy	Sequential adjuvant hormone therapy	Concomitant treatment	Specific clinical features	
1	70	pT1c	pN0	19	+	-	1	Right	Inferior Junction	No	Exemestane	No	Cardiovascular disease	
2	74	pT1c	pN1mi	13	+	+	2	Left	Central	No	Exemestane	No	Cardiovascular disease	Chronic respiratory insufficiency
3	72	pT1b	pN0	9	+	-	1	Left	SI	No	Letrozole	No	No	
4	67	pT1c	pN0	14	+	-	2	Right	Superior Junction	No	Tamoxifen	No	No	
5	78	pT1a	pN0	3	+	-	2	Right	SE	No	No	No	No	
6	44	pT1c	pN0	15	+	+	3	Right	SE	No	Letrozole	No	Major scoliosis	Chronic respiratory insufficiency
7 (right)	74	pT1c	pN0	17	+	-	1	Right	SI	No	Anastrozole	No	Chronic respiratory insufficiency	
7 (left)	74	pT1c	pN0	19	+	-	2	Left	II	No	Anastrozole	No	Chronic respiratory insufficiency	
8	76	pT1b	pN0	9	+	-	2	Left	SE	No	Tamoxifen	No	No	
9	85	pT1c	pN0	17	+	-	2	Left	SE	No	No	No	No	

*HR* hormone receptor, *HER2* Human epidermal growth factor 2 receptor, *SBR* Grade of breast cancer according to Scarff-Bloom-Richardson score, *SE* Supero-external quadrant, *SI* Supero-internal quadrant, *II* Infero-internal quadrant, *TNM* Tumour Node Metastasis status

### Acquisition and simulation

The patients underwent computed tomography (CT)-based virtual simulation (CT Simulator, General Electric, Cleveland, OH) with 2.5 mm thick slices obtained at 2.5 mm intervals. Patients were in supine position with arms above the head (arms and knee support, Sinmed, The Netherlands) and within a personalized immobilization device (Mold Care, Bebig). The isocenter was set in the middle of the surgical tumour bed.

### Contouring and volume definition

Target volumes and OAR were delineated on CT scan slices. The gross tumour volume and clinical target volume (CTV) were considered as equal and included the surgical bed, the surgical clips placed within the lumpectomy cavity, and/or the seroma. The planning target volume (PTV) was defined as an isotropic circumferential margin of 18 mm surrounding the CTV and excluded the first 5 mm under the skin surface, thoracic wall, ribs and pectoral muscles. OAR were automatically or manually contoured: ipsilateral and contralateral lungs, heart, ipsilateral and contralateral breast (using wires), thyroid, oesophagus, and humeral heads.

### Treatment planning using RapidArc®

A maximum dose rate of 600 MU/min and 6 MV photon beams were used. The optimization process started with the constraints obtained with the IMRT plans. RapidArc® (RA, Eclipse software version 10.0.28, Helios, Varian, Palo Alto, CA) was delivered with two partial coplanar arcs (less than 240° of rotation) sharing the same isocenter and optimized independently and simultaneously. These two arcs were delivered with opposite rotations (clockwise and counterclockwise) so that the off-treatment time between the two beams was minimized to about 25 s. The field size and collimator rotation were determined using the automatic tool from Eclipse software to encompass the PTV. The first clockwise arc used a 45° collimator rotation in order to avoid the tongue-and-groove effect. The second arc rotated counterclockwise with a collimator rotation of 360° - X° (X° corresponding to the rotation of the collimator for the first arc). To improve results, we modified optimization constraints and priority factors of RA plans during optimization. These parameters were modified with regard to the DVH results for each patient.

### Dose prescription, dose constraints, dosimetric evaluation and dose delivery

The radiotherapy was prescribed in fractions of 4.0 Gy “bis in die” (b.i.d.) over 5 consecutive days (40.0 Gy in total), with a minimal 6-hour interval between each fraction. 99 % and 95 % of PTV were to receive 38.0 Gy and 40.0 Gy, respectively. The total dose delivered wasn’t to exceed 110 % of the prescribed dose. Volumes receiving more than 110 % of the prescribed dose (D) were considered as “hot spots”. The homogeneity index (HI) was defined as: HI = (D2–D98 %)/D median. V_x%_ was defined as the proportion of the total structure volume that received x% of the prescribed dose. To limit the ipsilateral lung exposure, volumes receiving 20.0 Gy (V_20Gy_) had to account for less than 3 % of the total structure volume; less than 10 % and 20 % for V_10Gy_ and V_5Gy_, respectively. Regarding the contralateral lung exposure, respective thresholds for V_20Gy_, V_10Gy_ and V_5Gy_ were 1 %, 2 % and 3 % of the lung volume. Moreover, to limit heart exposure, V_20Gy_ and V_5Gy_ were not to exceed 1 % and 70 % of the heart volume. Finally, exposure of the ipsilateral breast was expressed as V_50%_ and V_100%_. The mean and maximal doses delivered to the contralateral breast were also determined.

Image-guided radiotherapy with cone-beam computed tomography (CBCT) using soft-tissue matching was performed before each fraction. The γ-index methodology was used to validate the planned delivery with a minimum of 95 % of the points meeting a 3 %/3 mm criterion.

### Toxicity assessment

A clinical examination was performed before APBI. Acute and late adverse events were assessed 1, 2, and 6 months after APBI completion, then every 6 months until 5 years, and then annually, according to the National Cancer Institute Common Terminology Criteria for Adverse Events (NCI-CTCAE) version 4.0. The main expected toxicities included: breast pain, breast oedema, skin erythema, skin desquamation, radiation pneumonitis, telangiectasia, fat necrosis, skin pigmentation, skin atrophy, and breast fibrosis.

## Results

### Treatment planning

Mean PTV and breast volumes were 100.0 cm^3^ (range 38.9 cm^3^ to 219.5 cm^3^) and 899.7 cm^3^ (range 390.7 cm^3^ to 1932.3 cm^3^), respectively. The mean dose encompassing 95 % of the PTV accounted for 99.7 % of the prescribed dose (range 99.4–99.9 %). Hot spots accounted for 0.3 % of the PTV (range 0.0–1.4 %). Mean HI was 5.6 % (range 4.0–8.5 %).

**Fig. 1:**
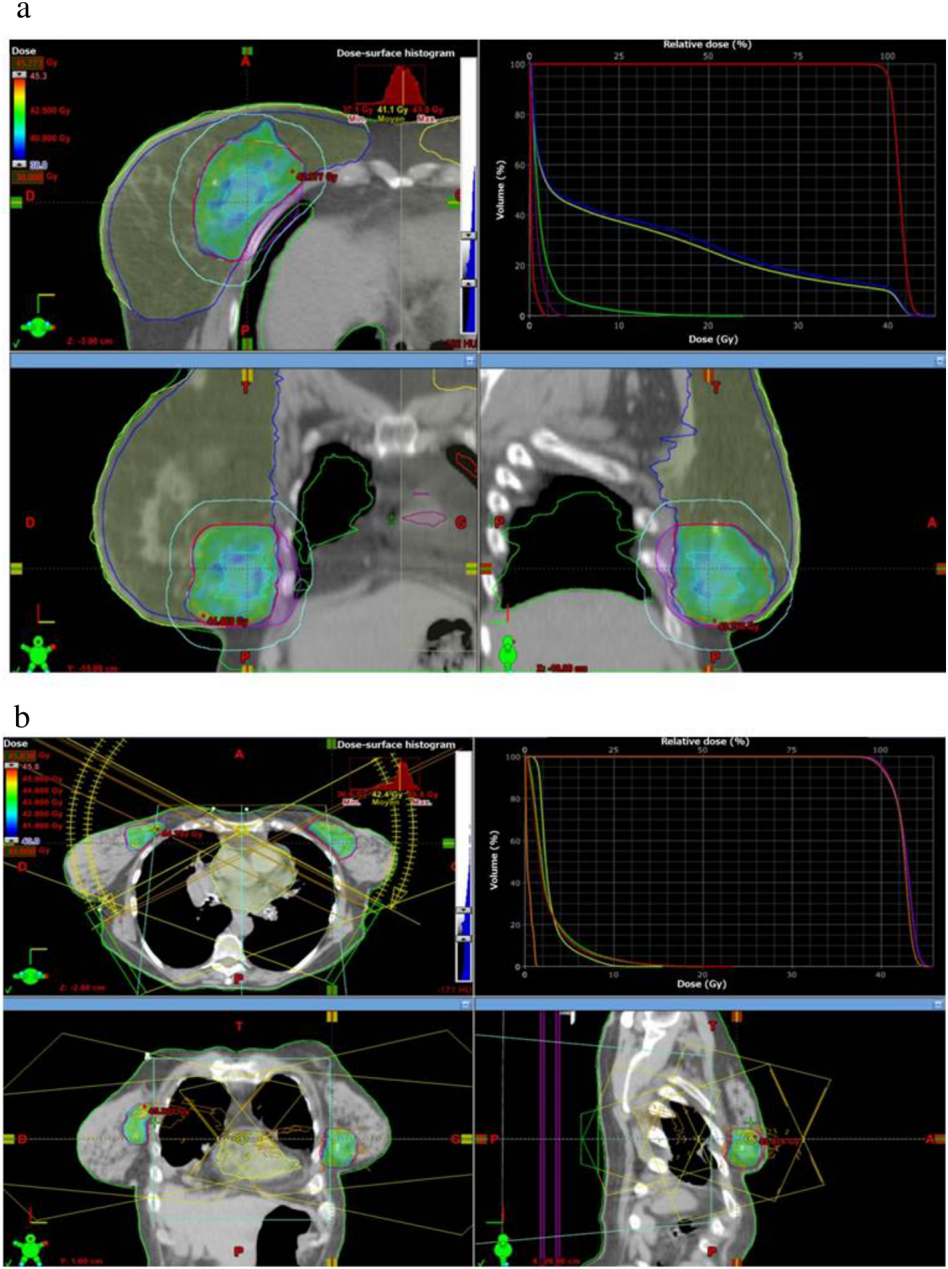
Typical dosimetric results obtained with accelerated partial breast irradiation using volumetric-modulated arc therapy on two patients: one right breast cancer (**a**) and one bilateral cancer (**b**). **a** (*upper left*) dose distribution in axial view, dose colorwash from 38.0 Gy to maximal dose at 45.3 Gy; (*upper right*) corresponding dose-volume histograms for this patient: planning target volume (PTV) in red, right breast minus 3 mm above the skin in blue, right breast in yellow, right lung in green, heart in purple, left lung in red; (*lower left*) dose distribution in coronal view, dose colorwash from 38.0 Gy to maximal dose at 45.3 Gy; (*lower right*) dose distribution in sagittal view, dose colorwash from 38.0 Gy to maximal dose at 45.3 Gy. **b** (*upper left*) dose distribution in axial view, dose colorwash from 40.0 Gy to maximal dose at 45.8 Gy; (*upper right*) corresponding DVH for this patient: left PTV in red, right PTV in purple, spinal cord in orange, heart in yellow, right lung in green, left lung in red; (*lower left*) dose distribution in coronal view, dose colorwash from 40.0 Gy to maximal dose at 45.8 Gy; (*lower right*) dose distribution in sagittal view, dose colorwash from 40.0 Gy to maximal dose at 45.8 Gy

**Fig. 2:**
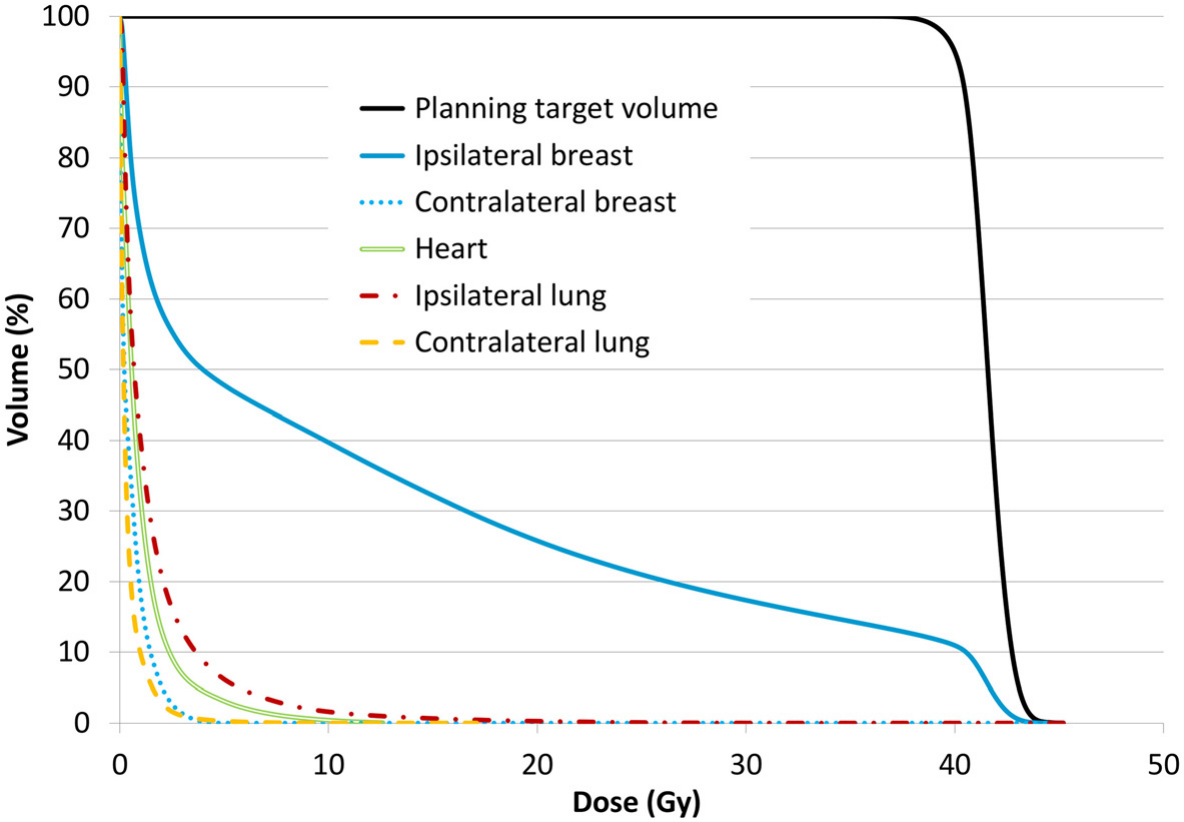
Mean dose-volume histograms for the ten lesions treated: planning target volume in violet, ispilateral breast in pink, contralateral breast in blue, homolateral lung in cyan, controlateral lung in yellow, heart in brown

The Fig. 1 shows typical dose distribution using RA for right breast cancer (a) and bilateral breast cancer (b). Mean dose-volume histograms are presented in Fig. 2 (n = 10). The main dosimetric results are presented in Table 2 for ipsilateral breast, contralateral breast, heart, and ipsilateral and contralateral lung. An average of 580 monitor units (MU) was delivered with RapidArc^®^ (range 473 MU to 655 MU). The mean treatment time was 3.2 min.

**Table 2:** Main dosimetric results regarding the protection of organs at risk

	Mean value	Range [min-max]
Ipsilateral breast (%)		
V_50%_	25.8	[13.3–37.8]
V_100%_	10.9	[5.6–18.9]
Contralateral breast (Gy)		
maximal dose	3.0	[1.3–5.8]
mean dose	0.6	[0.1–1.7]
Heart (%)		
V_5Gy_	3.1	[0.0–23.6]
Ipsilateral lung (%)		
V_5Gy_	6.2	[0.0–19.9]
V_10Gy_	1.6	[0.0–10.4]
V_20Gy_	0.3	[0.0–2.7]
Contralateral lung (%)		
V_5Gy_	0.3	[0.0–2.8]
V_10Gy_	0.0	
V_20Gy_	0.0	

V_x%_ proportion of the total structure volume that received x% of the prescribed dose, V_xGy_ proportion of the total structure volume that received x Gy

### Acute and late toxicity

As summarized in Table 3, acute toxicities were of grade 1 or less in all patients at any early time point (i.e. at APBI completion, 1 month and 2 months). Most observed symptoms were breast pain, oedema and erythema. There were no grade 2 or more adverse events. No treatment discontinuations occurred.

**Table 3:** Acute and late toxicities^a^ that occurred within three months after APBI with VMAT (acute) and up to 26 months of follow-up (late) (n = 10)

	Grade 0	Grade 1
Acute toxicities		
Breast pain	5	5
Breast oedema	5	5
Erythema	4	6
Dry desquamation	9	1
Moist desquamation	9	1
Radiation-induced pneumonitis	10	0
Late toxicities		
Breast pain	9	1
Breast oedema	10	0
Telangiectasia	10	0
Pigmentation	10	0
Fibrosis	7	3
Atrophy	10	0
Radiation-induced pneumonitis	10	0
Fat necrosis	10	0

*APBI* accelerated partial breast irradiation, *VMAT* volumetric-modulated arc therapy

^a^Toxicity was scored according to the National Cancer Institute common terminology criteria for adverse events version 4.0

Late toxicities were collected with a median follow-up of 26 months (range 6–37 months). Only three grade 1 fibrosis and one grade 1 breast pain were observed. No toxicities of greater grade were reported.

### Oncological outcomes

With a median follow-up of 26 months, no local or distant relapses were diagnosed.

## Discussion

VMAT for APBI has not been extensively studied in the literature. We present here the first feasibility study including clinical results in this setting. VMAT, as a rotational IMRT technique, has the potential to deliver highly conformal and homogeneous dose to the targeted volumes. However, because of its rotational nature, it is thought to be less efficient at low and medium doses, thereby irradiating a larger OAR volume. On the one hand in our study, such dispersion was not observed and we obtained a dose distribution to the ipsilateral lung, contralateral lung and heart similar to the one reported in 3-dimensional (3D) conformal APBI [16, 26]. On the other hand, we should remain aware of the low but not negligible dose delivered to the contralateral breast with this technique (Table 2). Indeed, this effect has not been reported when using tangential field 3D conformal APBI. Moreover, the ipsilateral breast exposure to a significant radiation dose seems to be a significant risk factor of unacceptable toxicity and impaired cosmesis [15]. The ipsilateral breast is considerably less irradiated with VMAT as compared to other external beam radiotherapy techniques: mean V_50%_ and V_100%_ being respectively of 25.8 % and 10.9 % in our study versus 47.9 % and 27.2 % in the IMRT study by Jagsi et al.[15], and 44.1 % and 23.8 % in the 3D-CRT study by Bourgier et al. [16, 26] Our results remain competitive when compared with those obtained in other studies on VMAT for APBI published by Qiu et al. [22] and Essers et al. [23].: they reported ipsilateral breast V_50%_ of 45.9 and 19.7 % and V_100%_ of 20.9 % and not reported, respectively. However, in our study, a greater maximal dose was delivered to the contralateral breast: 3.0 Gy [1.3–5.8 Gy] versus 2.56 Gy [0.46–4.83 Gy] in the study by Qiu et al., but Essers et al. reported a greater maximal dose of 4.6 Gy [0.1–9.4 Gy]. Regarding the ipsilateral lung, we report lower V_10Gy_ and V_20Gy_ than Qiu et al. (respectively 1.6 % [0.0 %–10.4 %] *vs*. 2.0 % [0.0 %–5.0 %] and 0.27 % [0.00 %–2.67 %] *vs*. 0.5 % [0.0 %–2.0 %]), but a higher V_5Gy_ (6.2 % [0.0 %–19.9 %] *vs.* 5.8 % [0.0 %–11.2 %]). Finally, Essers et al. reported a higher ipsilateral lung V_5Gy_ (10.4 % [0.0 %–40.3 %]). Another factor that might be involved in the onset of late fibrosis and retraction is the treated volume and/or the PTV to whole breast ratio. In the study by Jagsi et al., the mean PTV was 185.8 cm^3^ compared to 123 cm^3^ in the study by Livi et al., 117 cm^3^ in the study by Bourgier et al. and 100.0 cm^3^ in our study [17, 18]. These differences in PTV can be explained by different surgical and remodelling techniques, entailing large variations in surgical bed and seroma cavity volumes. Indeed, APBI may not be the most suitable method to treat patients with large lumpectomy cavities.

Finally, PTV homogeneity is believed to be an important factor for cosmetic results [27]. Our homogeneity index was 5.6 [4.0–8.5], which compares favourably to 3D APBI studies (e.g. 9.7 [6.2–15.1] in the study by Bourgier et al.).

Regarding exposure of OAR, especially lung, contralateral breast and heart, no comparison has yet been made between VMAT and static field IMRT. Whether the higher modulation possibilities of VMAT could improve OAR protection remains to be proven.

Acute toxicity was acceptable as all patients had grade ≤ 1 toxicities. With a median follow-up of 26 months, late toxicity was low, most of the patients experiencing none; and no grade 2 late toxicities occurred. Nevertheless, a longer follow-up and a larger cohort of patients are warranted to consider VMAT as a safe APBI modality.

Our study has some limitations, including the low number of patients and the limited follow-up time. This is mainly due to the fact that APBI is not a standard treatment outside clinical trials in France; therefore we mainly recruited patients presenting clinical features that would not allow standard radiotherapy, such as advanced age, respiratory insufficiency or cardiovascular disease. The treatment tolerance seemed to be especially good in this population. This limited follow-up allowed us to consider the clinical toxicity only without drawing any conclusion regarding tumour control. If longer-term studies confirm its efficacy and tolerability, VMAT APBI might become a therapeutic alternative for patients otherwise not treated.

## Conclusions

In this study performed in nine patients with breast cancer, VMAT offered a good OAR sparing while maintaining PTV coverage within acceptable levels for APBI. The early evaluation of oncological outcomes was promising.

## References

[1] Clarke M, Collins R, Darby S, Davies C, Elphinstone P, Evans E, Godwin J, Gray R, Hicks C, James S, MacKinnon E, McGale P, McHugh T, Peto R, Taylor C, Wang Y, Early Breast Cancer Trialists’ Collaborative Group (EBCTCG) (2005). Effects of radiotherapy and of differences in the extent of surgery for early breast cancer on local recurrence and 15-year survival: an overview of the randomised trials. Lancet.

[2] Fisher B, Anderson S, Bryant J, Margolese RG, Deutsch M, Fisher ER, Jeong J-H, Wolmark N (2002). Twenty-year follow-up of a randomized trial comparing total mastectomy, lumpectomy, and lumpectomy plus irradiation for the treatment of invasive breast cancer. N Engl J Med.

[3] Veronesi U, Cascinelli N, Mariani L, Greco M, Saccozzi R, Luini A, Aguilar M, Marubini E (2002). Twenty-year follow-up of a randomized study comparing breast-conserving surgery with radical mastectomy for early breast cancer. N Engl J Med.

[4] Lemanski C, Azria D, Gourgon-Bourgade S, Gutowski M, Rouanet P, Saint-Aubert B, Ailleres N, Fenoglietto P, Dubois J-B (2010). Intraoperative radiotherapy in early-stage breast cancer: results of the montpellier phase II trial. Int J Radiat Oncol Biol Phys.

[5] Polgár C, Major T, Fodor J, Sulyok Z, Somogyi A, Lövey K, Németh G, Kásler M (2010). Accelerated partial-breast irradiation using high-dose-rate interstitial brachytherapy: 12-year update of a prospective clinical study. Radiother Oncol.

[6] Njeh CF, Saunders MW, Langton CM (2012). Accelerated partial breast irradiation using external beam conformal radiation therapy: a review. Crit Rev Oncol Hematol.

[7] Vaidya JS, Joseph DJ, Tobias JS, Bulsara M, Wenz F, Saunders C, Alvarado M, Flyger HL, Massarut S, Eiermann W, Keshtgar M, Dewar J, Kraus-Tiefenbacher U, Sütterlin M, Esserman L, Holtveg HMR, Roncadin M, Pigorsch S, Metaxas M, Falzon M, Matthews A, Corica T, Williams NR, Baum M (2010). Targeted intraoperative radiotherapy versus whole breast radiotherapy for breast cancer (TARGIT-A trial): an international, prospective, randomised, non-inferiority phase 3 trial. Lancet.

[8] Vaidya JS, Wenz F, Bulsara M, Tobias JS, Joseph DJ, Keshtgar M, Flyger HL, Massarut S, Alvarado M, Saunders C, Eiermann W, Metaxas M, Sperk E, Sütterlin M, Brown D, Esserman L, Roncadin M, Thompson A, Dewar JA, Holtveg HMR, Pigorsch S, Falzon M, Harris E, Matthews A, Brew-Graves C, Potyka I, Corica T, Williams NR, Baum M, TARGIT trialists’ group (2014). Risk-adapted targeted intraoperative radiotherapy versus whole-breast radiotherapy for breast cancer: 5-year results for local control and overall survival from the TARGIT-A randomised trial. Lancet.

[9] Vaidya JS, Baum M, Tobias JS, Wenz F, Massarut S, Keshtgar M, Hilaris B, Saunders C, Williams NR, Brew-Graves C, Corica T, Roncadin M, Kraus-Tiefenbacher U, Sütterlin M, Bulsara M, Joseph D (2011). Long-term results of targeted intraoperative radiotherapy (Targit) boost during breast-conserving surgery. Int J Radiat Oncol Biol Phys.

[10] Chen PY, Wallace M, Mitchell C, Grills I, Kestin L, Fowler A, Martinez A, Vicini F (2010). Four-year efficacy, cosmesis, and toxicity using three-dimensional conformal external beam radiation therapy to deliver accelerated partial breast irradiation. Int J Radiat Oncol Biol Phys.

[11] Chafe S, Moughan J, McCormick B, Wong J, Pass H, Rabinovitch R, Arthur DW, Petersen I, White J, Vicini FA (2013). Late toxicity and patient self-assessment of breast appearance/satisfaction on RTOG 0319: a phase 2 trial of 3-dimensional conformal radiation therapy-accelerated partial breast irradiation following lumpectomy for stages I and II breast cancer. Int J Radiat Oncol Biol Phys.

[12] Formenti SC, Hsu H, Fenton-Kerimian M, Roses D, Guth A, Jozsef G, Goldberg JD, Dewyngaert JK (2012). Prone accelerated partial breast irradiation after breast-conserving surgery: five-year results of 100 patients. Int J Radiat Oncol Biol Phys.

[13] Azria D, Bourgier C (2010). Partial breast irradiation: new standard for selected patients. The Lancet.

[14] Smith GL, Xu Y, Buchholz TA, Giordano SH, Jiang J, Shih Y-CT, Smith BD (2012). Association between treatment with brachytherapy vs whole-breast irradiation and subsequent mastectomy, complications, and survival among older women with invasive breast cancer. JAMA.

[15] Jagsi R, Ben-David MA, Moran JM, Marsh RB, Griffith KA, Hayman JA, Pierce LJ (2010). Unacceptable cosmesis in a protocol investigating intensity-modulated radiotherapy with active breathing control for accelerated partial-breast irradiation. Int J Radiat Oncol Biol Phys.

[16] Bourgier C, Acevedo-Henao C, Dunant A, Rossier C, Levy A, Nemr ME, Dumas I, Delaloge S, Mathieu M-C, Garbay J-R, Taghian A, Marsiglia H (2012). Higher toxicity with 42 Gy in 10 fractions as a total dose for 3D-conformal accelerated partial breast irradiation: results from a dose escalation phase II trial. Radiat Oncol.

[17] Livi L, Buonamici FB, Simontacchi G, Scotti V, Fambrini M, Compagnucci A, Paiar F, Scoccianti S, Pallotta S, Detti B, Agresti B, Talamonti C, Mangoni M, Bianchi S, Cataliotti L, Marrazzo L, Bucciolini M, Biti G (2010). Accelerated Partial Breast Irradiation With IMRT: New Technical Approach and Interim Analysis of Acute Toxicity in a Phase III Randomized Clinical Trial. Int J Radiat Oncol Biol Phys.

[18] Livi L, Meattini I, Marrazzo L, Simontacchi G, Pallotta S, Saieva C, Paiar F, Scotti V, De Luca Cardillo C, Bastiani P, Orzalesi L, Casella D, Sanchez L, Nori J, Fambrini M, Bianchi S (2015). Accelerated partial breast irradiation using intensity-modulated radiotherapy versus whole breast irradiation: 5-year survival analysis of a phase 3 randomised controlled trial. Eur J Cancer.

[19] Vieillot S, Azria D, Lemanski C, Moscardo CL, Gourgou S, Dubois J-B, Aillères N, Fenoglietto P (2010). Plan comparison of volumetric-modulated arc therapy (RapidArc) and conventional intensity-modulated radiation therapy (IMRT) in anal canal cancer. Radiat Oncol.

[20] Riou O, Regnault de la Mothe P, Azria D, Aillères N, Dubois J-B, Fenoglietto P (2013). Simultaneous integrated boost plan comparison of volumetric-modulated arc therapy and sliding window intensity-modulated radiotherapy for whole pelvis irradiation of locally advanced prostate cancer. J Appl Clin Med Phys.

[21] Vieillot S, Azria D, Riou O, Moscardo CL, Dubois J-B, Aillères N, Fenoglietto P (2011). Bilateral kidney preservation by volumetric-modulated arc therapy (RapidArc) compared to conventional radiation therapy (3D-CRT) in pancreatic and bile duct malignancies. Radiat Oncol.

[22] Qiu J-J, Chang Z, Wu QJ, Yoo S, Horton J, Yin F-F (2010). Impact of volumetric modulated arc therapy technique on treatment with partial breast irradiation. Int J Radiat Oncol Biol Phys.

[23] Essers M, Osman SOS, Hol S, Donkers T, Poortmans PM (2014). Accelerated partial breast irradiation (APBI): are breath-hold and volumetric radiation therapy techniques useful?. Acta Oncol.

[24] Polgár C, Van Limbergen E, Pötter R, Kovács G, Polo A, Lyczek J, Hildebrandt G, Niehoff P, Guinot JL, Guedea F, Johansson B, Ott OJ, Major T, Strnad V (2010). Patient selection for accelerated partial-breast irradiation (APBI) after breast-conserving surgery: recommendations of the Groupe Européen de Curiethérapie-European Society for Therapeutic Radiology and Oncology (GEC-ESTRO) breast cancer working group based on clinical evidence (2009). Radiother Oncol.

[25] Smith BD, Arthur DW, Buchholz TA, Haffty BG, Hahn CA, Hardenbergh PH, Julian TB, Marks LB, Todor DA, Vicini FA, Whelan TJ, White J, Wo JY, Harris JR (2009). Accelerated partial breast irradiation consensus statement from the American Society for Radiation Oncology (ASTRO). Int J Radiat Oncol Biol Phys.

[26] Bourgier C, Pichenot C, Verstraet R, El NM, Heymann S, Biron B, Delaloge S, Mathieu M-C, Garbay J-R, Bourhis J, Taghian AG, Marsiglia H (2011). Early side effects of three-dimensional conformal external beam accelerated partial breast irradiation to a total dose of 40 Gy in one week (a phase II trial). Int J Radiat Oncol Biol Phys.

[27] Riou O, Fenoglietto P, Lemanski C, Azria D (2012). Radiothérapie conformationnelle avec modulation d’intensité dans les cancers du sein : intérêt, limitations, modalités techniques. Cancer/Radiothérapie.

